# Media, Trust in Government, and Risk Perception of COVID-19 in the Early Stage of Epidemic: An Analysis Based on Moderating Effect

**DOI:** 10.3390/healthcare9111597

**Published:** 2021-11-20

**Authors:** Tao Xu

**Affiliations:** College of Law and Political Science, Zhejiang Normal University, Jinhua 321004, China; xutao@zjnu.edu.cn

**Keywords:** media, trust in government, risk perception, moderating effect

## Abstract

Previous research has revealed that environmental, social, and cultural factors affect people’s risk perception of COVID-19, especially the influence of media and trust, while the dynamics of how they affect it is still not clear. Through the analysis of online survey data, this article shows that there are two opposed paths of action. Trust in the government will enhance people’s confidence in controlling COVID-19. It then moderates and decreases the effects of people’s level and frequency of concernon the risk perception (both cognition and worries) of COVID-19, on the contrary, obtaining information from unofficial channels also moderates and increases the effects of the people’s level and frequency of concern on the second dimension (worries) of risk perception of COVID-19 rather than the first dimension (cognition). These conclusions have important policy implications for the control of the COVID-19 epidemic all over the world.

## 1. Introduction

The outbreak of COVID-19 in Wuhan rapidly swept the world, it had profound impacts on the economy and society [1,2,3,4,5], and also threatened human health both physically and mentally [6,7]. Governments around the world have adopted measures including closing the border, quarantining, and maintaining social distancing to deal with it [8]. Studies showed that the trajectory of an infectious disease was often determined by the behavior of individuals, and the behavior was in turn related to individuals’ risk perception [9,10]. Experience from all over the world, especially in China, showed that how people perceived the risk of COVID-19 would directly affect their follow-up response behaviors. Higher levels of risk perception were found to be associated with higher intentions to engage in preventive behaviors [11,12,13], it meant that if they believed that the risk of COVID-19 was high and dangerous, they would cooperate with the government’s anti-epidemic behavior, strictly adhering to the restrictions and the government could control the widespread of it and restored the economy and society quickly. On the contrary, if they believed that COVID-19 was just as influenza, and they would react negatively, ignoring or delaying the governments’ rules and mingling in crowds in public places, at beaches, or in their homes [14], which might lead to the further spread of the infection and caused a wider epidemic. Therefore, it is significant to research people’s risk perception, explore its influencing factors, and the mechanisms behind it, for the society as a whole to formulate relevant coping strategies in a timely and effective manner.

Risk perceptions refer to people’s intuitive evaluations of hazards that they are or might be exposed to, including a multitude of undesirable effects that people are involved in [15,16]. Some theorists conceptualized risk perception into two types: personal risk perception and societal risk perception [17,18]. For individuals, it refers to the perception and understanding of various objective dangers in the outside world, it was intended as a probabilistic assessment, comparing the probability that an event occurred and the seriousness of the potential damage [14]. Obviously, even with the same external conditions or dangers, different people’s perceptions are different. What we are interested in is how an individual’s risk perception forms societal risk perception.

Some research has revealed that various factors such as demographic, social, and cultural factors influenced risk perception [19,20,21]. Other researchers found that voluntariness, personal ability to influence risks, familiarity with the hazard, and catastrophic potential provided useful information to consider in constructing their interpretation of risks [22,23,24]. However, these psychological explanations cannot explain why people perceive some risks, but ignore others.

The sociological analysis provided some further social insights. Some studies explored coping strategies from the perspective of organization and management [22,23], while others explored the formation and changes of people’s attitudes towards risk-bearing activities [25,26]. More theoretically, some researchers emphasized the social construction of risk interpretations and their relationship to knowledge acquisition, social interests, and cultural values [25,26,27]. These had good explanatory power for an individual’s risk perception, but how individual risk perception evolved to societal risk perception was still to be explained.

### 1.1. Social Amplification of Risk Model

Kasperson et al. proposed the social amplification of risk model (SARM) to explain how the risk is amplified from the individual to the society, they pointed out that events related to the hazards of the interaction of psychological, social, institutional, and cultural processes might heighten or attenuate personal and social perceptions of risk, and shape risky behaviors. Behavior, in turn, could produce secondary social or economic consequences that go far beyond direct harm to humans or the environment, including major indirect effects, such as liability, insurance costs, loss of trust in institutions, or alienation from community affairs [22,28].

For them, the amplification process began with the physical events or identification of adverse effects. Individuals or groups selected specific characteristics of these events or certain aspects of research results and interpreted them based on their understanding. These interpretations formed a message and conveyed it to others. People collected and responded to risk-related information, and acted as “amplification stations” through behavioral responses or communication. The amplification station could be an individual, a group, or an organization [28].

The perception and amplification process could be subdivided into eight steps namely, passing through attention filters, decoding of signals, drawing inferences, comparing the decoded messages with other messages, evaluating messages, forming specific beliefs, rationalizing a belief system, and forming a propensity to take corresponding actions. This cognitive process was further supplemented by emotional and subconscious processes filtering incoming messages and codetermining their evaluation [29,30]. The decoding and evaluation process determined the recipient’s choice of important information. On the one hand, if the decoded message was inconsistent with previous beliefs, the signals were ignored or weakened. On the other hand, if the information was attractive or consistent with previous beliefs, the signals were intensified [29]. In this source–receiver model, signals were sent from some source to some receiver. Clusters of signals formed messages that flowed from the source to the receiver, but typically the messages must first flow through intermediate transmitters. Each of the intermediate transmitters intensified or attenuated certain of the incoming signals. Thus, the cluster of signals leaving the source would be altered during the transmission to the ultimate receiver [31].

The social amplification of risk model (SARM) well explained how the risk of an individual evolved into the societal risk. It also revealed the importance of the dissemination of information to risk perception, while the information was usually transmitted through the media.

### 1.2. Media and Risk Perception

According to SARM, the media not only disseminated risk information but also amplified risk information. Therefore, there was a close connection between the media and risk. However, there were disputes over the relationship between the two in the related research field.

Some studies pointed out that the media exaggerated some risks and ignored others, sacrificing objectivity for sensationalism [32], the media often went for the individual opinion about different hazards, and also seemed to favor the more extreme reactions among those concerned. Some others revealed that media reports tended to concentrate on rare but dramatic hazards, and often failed to report more common but serious risks, such as motor vehicle accidents [33]. Therefore, the researcher got such an impression that media content was influenced by the real risks as calculated by scientists but biased towards the dramatic.

Social learning theory explains why people’s risk perception was affected by the media. Humans learned not only by doing but also by watching and hearing. This meant that everything we experienced, even “second-hand”, taught us something about the world. When it came to TV and risk perception, the images on TV distorted our worldview, making us unrealistically afraid, because the content of many programs was very violent and much more serious than the real world [34,35].

The impersonal impact hypothesis explained why there were discrepancies in the research on the media and risk perception. It stated that hazard information would have different impacts on the perceived societal and personal risk levels. Any person could be influenced by the media to feel that the risk for society was larger than previously thought, but this heightened risk judgment would not greatly affect personal risk judgment [36].

Gerbner et.al proposed the cultivation theory indicating that people would grow more fearful with a heightened amount of TV viewing, as they would see the world more as it was on the screen than it was in reality [37,38]. The logic behind it was that the more you watch TV, the more you lost your grasp of reality, and you just used available information to form your own judgment. While it was challenged by later research [39,40]. As for the connection between risk perception and anxiety, whether anxiety was caused by media exposure is uncertain, on the contrary, that it is not a predictor of behavior related to the anxiety object [41].

### 1.3. Trust in Government and Risk Perception

Studies have shown that trust was the most crucial factor affecting people’s risk perception of a specific hazard [42,43], and trust could be categorized as social and generalized trust. The former referred to trust in those whom people did not personally know or in institutions responsible for regulating or handling certain hazards [42,43]. While the latter referred to the differing characteristics between individuals concerning their willingness to trust other members of society in general [42,44,45]. Some researchers explored the relationship between trust and risk perception from the perspective of risk management and indicated that when people lacked knowledge, they must rely on trusted institutions to assess the risk of hazards. The similarity heuristic was considered to be the mechanism for the close link between trust and risk perception [46,47,48]. Infectious diseases like COVID-19 were a public hazard, and the governments had to deal with it by providing information concerning preventive behaviors to enhance people’s risk perception. Further research showed that trust in the central government and trust in local governments had different impacts on risk perception. Trust in local governments had a greater impact on risk perception than trust in the central government because, under normal circumstances, the specific crisis was resolved by the local government [49].

Although there is controversy about the size of the effects of trust in different levels of government on risk perception, and the mechanism behind it, the researches recognized that trust in the government affected the perception of the public.

### 1.4. The New Framework of Risk Perception of COVID-19

A very important reason for the diametrically opposite conclusions of past research on risk perception is the vague or inconsistent definition of risk perception. In this article, two dimensions of risk perception of COVID-19 are proposed to define the risk perception. One dimension is the people’s judgment on the seriousness of COVID-19, which is the cognition of the risk perception, the other dimension is the hazard resulting in worries to be infected in the future.

**Figure 1:**
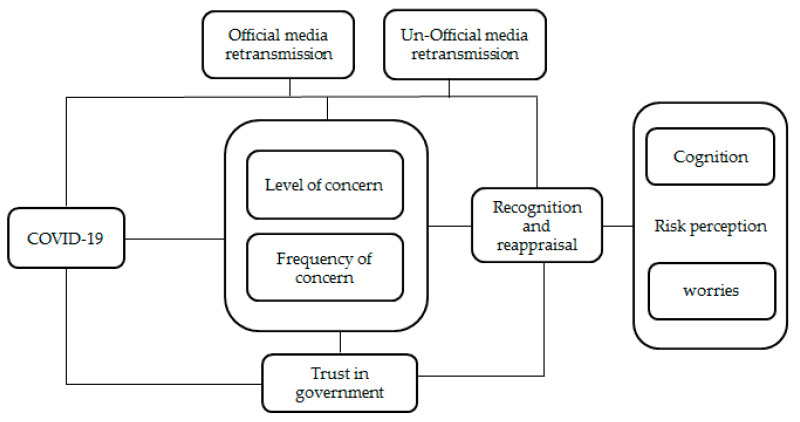
The new framework of risk perception of COVID-19.

Based on previous studies on the social amplification risk model and trust, confidence, and cooperation model (TCC), this article proposes a comprehensive analytical framework of risk perception of COVID-19, which includes both the amplification and the reduction dynamics of risk perception(Figure 1). We expect that the spread of risk perception may not only be amplified by media but also reduced by the trust in government.

Previous studies showed that personal risk perceptions would be amplified through the transmission of risk information by the media, however, most of the researches ignored the ways that different types of media transmitted information. Xu and Usman pointed out that media at least could be categorized into two types of media, namely official and unofficial media, and they played different roles in the spread of information. Unofficial channels spread a great deal of false, inaccurate, distorted, or even fabricated information. While timely disclosure of information by official channels could keep people informed of the status of the outbreak, and stabilize relevant psychological expectations and correct the inaccurate, distorted, and twisted parts of the information transmitted through the grapevine. It allowed people to fully understand accurate information, thus helping them to avoid groundless panic [50].

This meant that when people used different media to obtain information, the results of information dissemination might be different. During the COVID-19 epidemic, to maintain social stability and people’s emotions, when releasing information related to the epidemic, the government would be very cautious and not exaggerate arbitrarily, and the information that people obtained from the government might not aggravate people’s perception of risk. Meanwhile, unofficial channels might exaggerate information related to the epidemic to catch eyes or seek commercial interests, thereby deepening people’s judgment on the risk of the epidemic.

In this situation, with the increase of the frequency and level of concern about COVID-19, on the one hand, their cognition on the risk perception of COVID-19 could be heightened for the ordinary people, on the other hand, by the volume of unconfirmed information about the serious situation of COVID-19, their worries of themselves, their family members and friends to be infected would increase accordingly. This indicated that probably unofficial information acquisition media moderated people’s attention level and frequency to COVID-19 and increased people’s risk perception especially the worries of infections.

**Hypothesis** **1:** *Different media played different roles in the spread of information, unofficial media information acquisition moderated and increased people’s level and frequency of concern about COVID-19 on people’s cognition and worries of risk perception*.

Researchers proposed the trust, confidence, and cooperation (TCC) model to assume that trust and confidence influenced the willingness to cooperate [46,47,48]. This model indicated that trust was based on the judgment of similarities in intentions and value [43].

COVID-19 was an infectious disease threatening the whole society including ordinary people and the government, both of them faced the same hazard. The responsibility of the government was to reduce the impacts of pandemics on people’s lives, they would attempt to provide a variety of information concerning preventive behaviors to ordinary people for reducing infection rates and controlling the extensive spread of COVID-19, such as wearing the mask, washing hands, and maintaining social distancing. Given that the people trusted in the government, they would be confident with the government and took preventive behaviors following the guidelines of the government, in this sense, it was easier to control the spread of the epidemic with their joint efforts, and the ordinary people’s worries to be infected would probably be lowered. Under this situation, even with the increase of the frequency and level of concern about COVID-19, ordinary people’s risk perception especially their worries of infections would not necessarily be heightened.

**Hypothesis** **2:** *Trust in government moderated and lowered the effects of people’s concern level and frequency of COVID-19 on the people’s risk perception (both cognition and worries)*.

## 2. Materials and Methods

### 2.1. Data

The data used in this study come from the Online Survey of Public Perception of COVID-19 and Its Social Consequences in 2020, which was mainly aimed at understanding people’s perception of COVID-19 and its consequences after the outbreak of the epidemic. This survey was conducted in February and lasted for one week, eventually obtaining 1613 cases, covering almost all regions in northeastern, northern, eastern, central, southern, southwestern, and northwestern China. In order to gain public perception and the consequences of COVID-19 in a short period, this survey adopted an online survey with the snowball method [51]. Specifically, a QR code of the questionnaire through WeChat was generated and released to research group members’ WeChat group and QQ friend circles and further disseminated through these friend circles. Volunteers were recruited to fill the survey questionnaire, before filling, a short consent letter was provided to inform them of the purpose of the survey and that all information provided would be strictly protected according to the law. After deleting 76 missing surveys, because more than one-third of questions were not answered, finally, 1537 responses were used [50]. All data were processed by Stata 12.0.

### 2.2. Measures

#### 2.2.1. Dependent Variable

*Risk Perception of COVID-19.* Based on previous research [6,52,53,54], following Dryhurst et al. [55], our dependent variable “Risk Perception of COVID-19” was measured as two indexes, covering cognitive and affective dimensions to provide an integrated measure of risk perception. Coincidentally, some other scholars also proposed two other dimensions: dread and risk of the unknown to measure risk perception [24,56]. In this article, the cognitive dimension mainly referred to people’s cognition of the COVID-19, while the affective dimension mainly related to people’s worries of infections of COVID-19.

In the questionnaire, a set of subjective evaluation items that scale the risk perception of COVID-19 were included. The index included items capturing participants’ cognition on the seriousness of the COVID-19, relative items are as follows: “How do you agree that COVID-19 is highly infected/rapid fatality/high mortality?” The variables are measured on an ordinal scale with 5 possible answers: strongly disagree, disagree, neutral, agree, and strongly agree, coded as 1 to 5. The sum score of these 3 items was as the first index (cognition) of risk perception of COVID-19 (Cronbach’s alpha = 0.7887, *p* < 0.000).

Also, there were some other items on the evaluation of possible infection in the future, e.g., “How likely do you think it is that yourself/your friends and family/ordinary citizen will be directly affected in the future?” Alternative answers arranged from not likely to very likely in 5 equal intervals, also coded as 1 to 5. The result showed that it was reliable to use this scale to measure the risk perception of COVID-19 (Cronbach’s alpha = 0.7889, *p* < 0.000). We summed the score and used it as the second index (worries) of risk perception of COVID-19.

#### 2.2.2. Independent Variable

*Having COVID patients or not in their community.* This variable mainly asked whether there were COVID patients in the community, the answers were yes and no, we coded yes 1, and no 0.

*Level of concern about COVID-19.* This mainly asked respondents what kind of level of concern they had about COVID-19, the answers were arranged as not concerned at all, not concerned, general, concerned, very concerned, we coded them from 1 to 5, respectively.

*Frequency of concern about COVID-19.* Concerning the external situation of the outbreak, the survey mainly checked on the frequency of attention paid to COVID-19 through media. The answers on the frequency of attention ranged from not concerned, once every few days, once a day, twice a day, 3–5 times a day, and more than 5 times a day, coded from 1 to 5.

#### 2.2.3. Moderating Variable

*Trust in government.* There was a question “How much do you trust in the government to deal effectively with the pandemic”, the alternative answers were arranged as not trust at all, not trust, not trust a little, trust a little, trust, and very trust. In order to explore the moderating role of it through statistical analysis, we combined the first 3 and latter 2 answers, respectively, and then coded them into 0 and 1.

*Obtaining information channels.* Channels used by respondents to obtain information including Xinhua news agency, CCTV, People’s Daily mainstream media, traditional newspapers, magazines, official microblogs, official WeChat accounts, commercial accounts, microblogs, WeChat circles, personal microblogs, Douyin, Kuaishou, and other short videos, relatives, friends, colleagues, etc. Official TV stations, newspapers, official microblogs, and official WeChat accounts were taken as the official channels, and personal microblogs, WeChat circles, Douyin, or individuals’ relatives, friends, colleagues, etc were considered as the unofficial channels.

#### 2.2.4. Control Variable

*Gender* was used as a dichotomous variable (male coded as 0, female coded as 1). *Education* level was measured as an ordinal variable (primary school or below, junior high school, high school, college, master’s degree, or above). *Age* was measured as a ratio variable, and age squared was created as a control variable. *Marital status* including single, married, divorced, and widowed, was used as a categorical variable, and single was used as the referenced group.

### 2.3. Data Analysis Strategy

This study used the linear regression equation model [57,58] and the OLS (ordinary least squares) method [59] to analyze people’s risk perception of COVID-19. The specific expression is as follows:

Y = β_0_ + β_1 × 1_ + β_2_X_2_ + …… + β_k_X_n_ + ε

Y is the dependent variable of risk perception of COVID-19, X is the various independent variables that may affect people’s risk perception of COVID-19, and β is the coefficient.

In order to test the robustness of the model results, we reconstructed the dependent variable again using exploratory factor analysis and then rebuilt all the models. By comparison between the models’ results, we tested their robustness.

## 3. Results

### 3.1. Descriptive Statistics

From Table 1, we could see that there were 471 males in the sample, representing approximately 30.67%, and 1067 females, representing approximately 69.33%, and the chi^2^ test showed that there was a significant difference in the first dimension (cognition) of risk perception but no difference in the second dimension (worries) of risk perception of COVID-19 between them (*p* < 0.01). In terms of education, only 3 belonged to the primary school and below group, 29 junior school, 82 senior high school, 970 were undergraduates, and 455 were postgraduates. Respectively, the percentages were 0.19%, 1.88%, 5.33%, 60.03%, and 20.06%, while the chi2 test showed that there were no significant risk perception differences between people in different education levels (*p* > 0.05). Meanwhile, for marital status, 1097 were single, 420 were married, 18 were divorced, and 4 were widowed, and there was a significant difference in the first dimension (cognition) but no second dimension (worries) of the risk perception among different marital statuses (*p* < 0.05). At the same time, in terms of trust in the government, 1314, approximately 85.38%, trust in the government, and only 225, about 14.62%, of the people did not trust the government, and there was a significant difference both in the two dimensions of risk perception between them (*p* < 0.000). Furthermore, 163 people, about 10.59%, reported that there were COVID-19 patients in their community, and 1376, about 89.41%, reported no, and there was a significant difference in the second dimension of risk perception (worries) between them (*p* < 0.000). Lastly, 1239, about 80.51%, of the people reported that they obtained COVID-19 information from official media, and 300, about 12.49%, of the people reported they never got information from official channel, simultaneously, 931, about 60.49%, of the people reported that they obtained COVID-19 information from the unofficial channel, and 608, about 39.51%, of the people reported they never got information from the unofficial channel, and chi2 tests showed that there was a significant difference in the second dimension (worries) but no difference in the first dimension (cognition) of risk perception between the people using an unofficial channel or not (*p* < 0.01), while on the contrary, there was a significant difference in the first dimension (cognition) but no difference in the second dimension (worries) between the people using an official channel or not (*p* > 0.05).

To estimate people’s risk perception about COVID-19, we summed the first 3 items used as the indicator of the first dimension of risk perception (cognition) and summed the latter 3 items as the indicator of the second dimension of risk perception (worries), and found that the mean sum value of cognition was approximately 10.26 and mean sum value of worries was 12.43 (Table 1). Simultaneously, in order to explore the possible relationships between the scale variables and risk perception of COVID-19, such as level of concern about COVID-19, frequency of concern about COVID-19, we performed correlation analysis(Table 2). The results showed that there were close relationships between these two variables and the second dimension (worries) of risk perception of COVID-19 (*p* < 0.001), and a positive relationship between the first dimension (cognition) and frequency of concern about COVID-19 as well.

### 3.2. Regression Results

In order to distinguish the influences of multiple factors such as demographic variables, trust in government, having COVID patients or not in the community, obtaining information from the official channel and unofficial channel, frequency of paying attention to COVID-19 epidemic, and level of concern about COVID-19 on risk perception of COVID-19, and the specific affecting path of risk perception, we constructed five models. Model 1a and 2a examined the influence of trust in government, and obtaining information from the official channel and unofficial channel on the two dimensions of risk perception of COVID-19. Models 1b and 2b explored the effect of the interaction of obtaining information from the unofficial channel and level of concern about COVID-19 on people’s risk perception of COVID-19. Models 1c and 2c explored the effect of the interaction of obtaining information from unofficial channels and frequency of concern about the COVID-19 epidemic on people’s risk perception of COVID-19. Models 1d and 2d explored the influence of the interaction of trust in government and level of concern about COVID-19 on people’s risk perception of COVID-19. Models 1e and 2e tested the influence of the interaction of trust in government and the frequency of concern about COVID-19 on people’s risk perception of COVID-19.

In Table 3, the results of Model 1a and 2a showed that trust in the government was closely related to people’s risk perception of COVID-19 (both cognition and worries), if people trusted the government, their risk perception (both cognition and worries) would decrease, and vice versa. Simultaneously, concern for COVID-19 including the level of concern about COVID-19 and frequency of paying attention to the epidemic were closely related to people’s second dimension of risk perception (worries) (*p* < 0.000), while only the level of concern was closely related to the first dimension (cognition) of risk perception, and frequency of concern about COVID-19 did not have a similar effect. Lastly, obtaining information from the unofficial channel was positively related to the second dimension of risk perception (worries) while not related to the first dimension (cognition), on the contrary, there was a negative association between obtaining information from the official channel and the first dimension of risk perception (cognition), while there was no connection between obtaining information from the official channel and the second dimension of risk perception.

Concern level about the COVID-19 epidemic was closely related to the risk perception of COVID-19. In order to examine the moderating effect of channels for obtaining information on the concern of COVID-19, we constructed Models 1b, 2b, 1c, and 2c.

The results of Model 1b and 1c showed both the interaction of obtaining information from the unofficial channel and frequency of concern about COVID-19 and the interaction of obtaining information from unofficial channel and level of concern about COVID-19 did not affect the first dimension of people’s risk perception (cognition). However, Models 2b and 2c showed that the interaction of obtaining information from the unofficial channel and level of concern about COVID-19 positively affected the second dimension of people’s risk perception (worries) as well as the interaction of obtaining information from unofficial channel and frequency of concern about COVID-19, which meant that once people utilized unofficial channels to obtain information about COVID-19, as their attention level and frequency increased, their perceived risks (worries of infection) became heightened.

The results of Model 1b, 2b, 1c, and 2c showed that unofficial information acquisition channels moderated and increased the effects of people’s attention level and frequency to COVID-19 on people’s worries of infection (the second dimension of risk perception), while did not affect the first dimension of risk perception (cognition). This indicated that Hypothesis 1 was partially confirmed and also revealed that too much redundant information was not conducive to our correct understanding of the COVID-19 epidemic.

For the same purpose as Models 1b, 1c, 2b, and 2c, in order to investigate the moderating effect of trust in government on the concern of COVID-19, we constructed Models 1d, 1e,2d, and 2e. The results of Models 1d and 2d showed that interaction of trust in government and level of concern about COVID-19 negatively affected both the two dimensions of people’s risk perception (cognition and worries), which meant that if people trusted their government, as their attention level increased, their perceived risks (both cognition and worries) decreased. Simultaneously, the results of Models 1e and 2e showed that that interaction of trust in government and frequency of concern about COVID-19 negatively affected both two dimensions of people’s risk perception (cognition and worries), which meant that if people trusted their government, as their attention frequency increased, their perceived risks (both cognition and worries) decreased as well.

The above results showed that obtaining information from unofficial information channels moderated people’s attention frequency and level to COVID-19, which in turn increased the second dimension of people’s risk perception of COVID-19 (worries). Conversely, people’s trust in the government also moderated people’s attention frequency and level to the COVID-19, however, it could significantly reduce the two dimensions of people’s perception of risk (cognition and worries). Hypothesis 2 was confirmed.

### 3.3. Robustness Analysis

**Figure 2:**
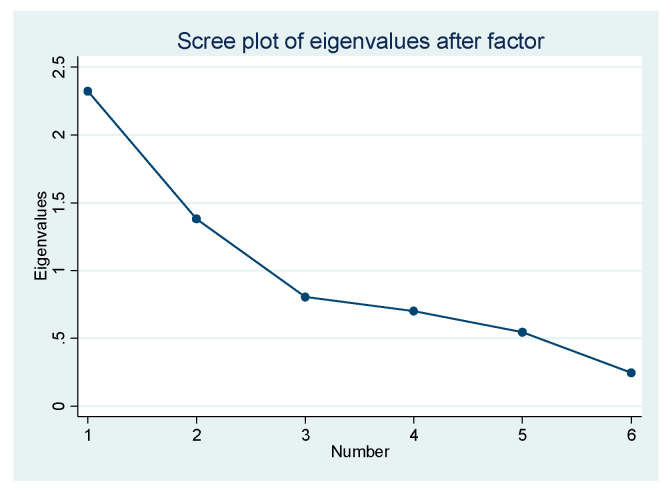
Scree plot of exploratory factor analysis.

In order to test the robustness of the above analytical results, we used another method to construct the dependent variable, risk perception of COVID-19, and then rebuilt the models again. We sought to extract the factors associated with the six items of the scale by using exploratory factor analysis. The reliability was good and the internal consistency was also high (Cronbach’s alpha = 0.773), and the Kaiser–Meyer–Olkin value was 0.8653 (*p* < 0.000), which indicated that these items were suitable for exploratory factor analysis. Therefore, we conducted exploratory factor analysis by principal component and varimax rotation. From the results of Table 4 and Figure 2, we can see that only the eigenvalue of factor 1 was larger than 1, which showed that two factors could be extracted. Thus, we extracted these two factors and named the first factor as cognition of risk perception, and the second factor as worries about infection of COVID-19 according to the correlation of the indicators and their meanings. The factor scores were respectively saved as the dependent variables.

With the same purpose as the previous models, to explore the moderating role of the obtaining information channel and trust in government, as well as to confirm the robustness of the previous results, we established another five models.

We rebuilt all the models (3a–3e and 4a–4e) as in the previous analysis (Table 5). On the one hand, the results confirmed again that the higher level the concern as well as the more frequent concern, the higher level of perceived risk. Simultaneously, the results not only confirmed that obtaining information from unofficial information channels moderated the effects of people’s concern frequency and level on people’s second dimension of risk perception of COVID-19 (worries) but also showed the moderating effects of obtaining information from unofficial information channels on the first dimension of risk perception (cognition). On the other hand, it also showed that people’s trust in the government moderated the effects of people’s concern frequency and level on both the two dimensions of people’s perception of risk (cognition and worries). The results were almost consistent with those of the former models except for a little nuance.

Considering that the dependent variable was subjective, and there were also some subjective variables in the explanatory variables, to get robust results, we also performed quantile regression with 0.25, 0.50, and 0.75. The results show there was no substantial change for the significant level of the explanatory variables (due to space limitations, the relevant results are not reported here, and can be obtained by email). Therefore, the results were robust.

More directly and robustly to show the results, we made Table 6 based on the results of the previous model and testing models. It showed that obtaining information from unofficial media increased the second dimension of risk perception (worries) while obtaining information from official media decreased the first dimension of risk perception (cognition). Furthermore, the level of concern about COVID-19 increased both the dimensions of risk perception, while the frequency of concern only increased the second dimension of risk perception (worries). Trust in government moderated and decreased the effects of people’s level and frequency of concern about COVID-19 on the risk perception (both cognition and worries) of COVID-19, while obtaining information from unofficial channels moderated and increased the effects of the people’s level and frequency of concern on the second dimension (worries) of risk perception of COVID-19 rather than the first dimension (cognition).

## 4. Discussion

In this article, we discussed the relationships between trust in government, media use, concern level and frequency of COVID-19, and the risk perception of COVID-19. On the one hand, the higher the people’s level of concern about COVID-19, the more worries they perceived (higher second the dimension of risk perception), simultaneously, the more frequently they paid attention to COVID-19, the higher risk perception (both cognition and worries) they attained, which is consistent with the previous related research [50,60].

On the other hand, in previous research [14,55], the effect of trust in government on risk perception of infectious diseases such as SARS, MERS, and even COVID-19 was confirmed. While in this article, the moderating role of trust in government was found. Specifically, citizens’ trust in government significantly moderated the effects of level and frequency of attention paid to COVID-19 on risk perception (both cognition and worries). The mechanism is that the attention to the COVID-19 epidemic usually significantly increased the judgment of the risk of the COVID-19, and this would further bring psychological pressure and cause psychological problems. However, the people’s trust in the government to effectively control the epidemic strengthened the confidence of the whole society in conquering and controlling COVID-19, thereby alleviating their mental pressure. Therefore, trust in the government was a manifestation of confidence and belief, and holding this belief, even if they increased their level and frequency of attention to COVID-19, it would not significantly increase their judgment of the risk of the COVID-19 epidemic because they had inherent confidence and belief in the government to control the epidemic. In this sense, people’s trust in the government could not only directly reduce people’s worries about risks but also moderate other variables to indirectly reduce people’s worries about risks, thereby reducing the probability of people’s mental and psychological illness due to the COVID-19 pandemic.

At the same time, we also had another important finding, contrary to the moderating effect of trust in the government, obtaining information through unofficial channels was an important moderating variable that amplified risk perception (both cognition and worries). Specifically, on the one hand, if people mainly obtained information through unofficial channels, due to the lack of control by unofficial media, and to expand followers and make profits, they might arbitrarily attract people by expanding the severity and consequences of the real epidemic [50]. In this context, once the frequency and level of people’s attention to the COVID-19 epidemic increased, through the invisible mechanism of the unofficial media, people’s awareness of risks would be increased. On the other hand, this increased risk awareness would be passed through the unofficial media. The reprocessing and magnification of the disease spread to other groups of people, which in turn increased the risk perception of the entire society and caused psychological problems. These conclusions not only confirmed again the negative role of the unofficial channel in amplifying the COVID-19 epidemic, which was consistent with previous research [30,50,61,62] but also explored the moderating role of the unofficial channel which contributed to helping us more clearly understand the hidden mechanism of judgment on the increased risk of the COVID-19, in turn improving our response to it.

## 5. Conclusions

In addition to environmental, social, and cultural factors that affect people’s risk perception of COVID-19, there were two opposed opposite mechanisms for the impact of trust in government and obtaining information channel on risk perception of COVID-19. Trust in the government would enhance people’s confidence in controlling COVID-19. It then moderated and decreased the effects of people’s level and frequency of concern about COVID-19 on the risk perception (both cognition and worries) of COVID-19, on the contrary, obtaining information from unofficial channels also moderated and increased the effects of the people’s level and frequency of concern about the second dimension (worries) of risk perception of COVID-19 rather than the first dimension (cognition). The contribution of this research is to divide risk perception into cognitive (cognition) and affective dimensions (worries) of risk perception and explore the differences in the moderating impacts of media and trust in government on the different dimensions of risk perception.

This research also had some limitations. Firstly, the data in this study was completed through online surveys in a short time. Due to the overall unclearness and unclear boundaries of the online survey method itself, it was likely that the representativeness of this study had certain problems. For example, in the sample of this study, there was a gap in the gender ratio between men and women, and the respondent’s education level was relatively high. These probably influenced the conclusions to some extent, while they were not fatal to this study, as these two indicators were not the core explained variables but just control variables and the conclusions drawn by this study were almost consistent with the previous research. Secondly, some studies revealed that values might be an important variable that affects people’s risk perception as well as different ideologies in different countries, and this research did not pay attention to it due to the limit of the data. What is more, some studies explored that trust in different levels of government may have different effects on the risk of perception [49,63], while these studies did not make a detailed comparison between them either, in the following research it should be focused on. Simultaneously, with the increasing of the related investigations and even cross-border surveys all over the world, we can compare the differences of people’s risk perception of COVID-19 among countries with different epidemics in the future and explore the similarities and differences on the affecting factors and mechanism. Thirdly, this article only focuses on people’s risk perception of COVID-19 in the early stage. Over time, whether people’s risk awareness will undergo subversive changes requires further research. Finally, according to the theory of planned behavior [64], the perception of risk will affect subsequent behaviors. Therefore, what kind of protective behaviors will be brought about, and what kind of risk perception will be transformed into proactive and effective behavior is still worthy of future research.

## 6. Policy Implications

These conclusions have some important implications for the control of the COVID-19 epidemic. Firstly, due to the positive effect of trust in government, during the epidemic, citizens should have confidence and trust in their governments and follow the preventive guidelines of the governments, then the governments can unite the power of the whole society to effectively respond to and control the spread of COVID-19. Secondly, due to the negative effects of unofficial media, all governments should grasp the release system of epidemic-related information, and strengthen control over the dissemination and spread of unidentified epidemic-related information by non-official media. Thirdly, given the scientific awareness of COVID-19, it not only helps us to prevent COVID-19 but also enables the public to maintain a good mentality without causing excessive worries. Therefore, it is very necessary to popularize the scientific and technological knowledge of COVID-19 and its prevention to the public. However, we have to admit that the most effective response to COVID-19 and the most effective way to deal with the worries caused by the COVID-19 epidemic still depends on the advancement of science and technology.

## Figures and Tables

**Table 1:** Description of the data.

TitleVariables	Frequency	Percentages	Cognition	Significance	Worries	Significance
Sex				*p* < 0.05		*p* > 0.05
Male	471	30.67	10.01		12.37	
Female	1067	69.33	10.37		12.45	
Education level				*p* > 0.05		*p* > 0.05
Primary school and below	3	0.19	11.00		13.00	
Junior school	29	1.88	10.86		12.86	
Senior school	82	5.33	10.96		12.79	
College	970	60.03	10.08		12.34	
Postgraduate	455	20.06	10.45		12.51	
Marital status				*p* < 0.01		*p* > 0.05
Single	1097	71.28	9.92		12.26	
Married	420	27.29	11.07		12.83	
Divorced	18	1.17	12.22		12.89	
Widowed	4	0.26	9.75		12.25	
Trust in government				*p* < 0.001		*p* < 0.001
Yes	1314	85.38	10.19		12.33	
No	225	14.62	10.63		12.98	
Having COVID patients or not in their community				*p* < 0.05		*p* < 0.05
Yes	163	10.59	10.17		12.44	
No	1376	89.41	10.297		12.42	
Obtaining information from the official channel				*p* < 0.001		*p* > 0.05
Yes	1239	80.51	10.19		12.42	
No	300	19.49	10.51		12.43	
Obtaining information from the unofficial channel				*p* > 0.05		*p* < 0.05
Yes	931	60.49	10.40		12.17	
No	608	39.51	10.04		12.59	
	**Mean**	**Std Dev**				
Cognition	10.26	2.54				
Worries	12.43	1.76				
Level of concern about COVID-19	4.53	0.61				
Frequency of concern about COVID-19	5.35	0.87				
Age	26.71	9.46				

**Table 2:** Correlation matrix.

	Cognition	Worries	Level of Concern about COVID-19	Frequency of Concern about COVID-19
Cognition	1.0000			
Worries	0.2138 ***	1.0000		
Level of concern about COVID-19	0.0166	0.3304 ***	1.0000	
Frequency of concern about COVID-19	0.0380 ***	0.2645 ***	0.4115 ***	1.0000

*** *p* < 0.01, ** *p* < 0.05, * *p* < 0.1.

**Table 3:** Regression model of trust in government and obtaining information channels.

Variables	Model 1a	Model 1b	Model 1c	Model 1d	Model 1e	Model 2a	Model 2b	Model 2c	Model 2d	Model 2e
Frequency of concern (FC)	−0.0205	−0.0283	−0.0208	−0.0207	0.0447	0.405 ***	0.378 ***	0.404 ***	0.405 ***	0.494 ***
Level of concern (LC)	0.443 ***	0.444 ***	0.435 ***	0.508 ***	0.443 ***	0.716 ***	0.716 ***	0.695 ***	0.803 ***	0.715 ***
Trust in government (TG) (No = 0)	−0.439 ***	−0.441 ***	−0.440 ***			−0.584 ***	−0.586 ***	−0.586 ***		
Obtaining information from official media (OIFOM)	−0.353 **	−0.353 **	−0.353 **	−0.353 **	−0.353 **	−0.0103	−0.00887	−0.0101	−0.0102	−0.0111
Obtaining information from unofficial media (OIFUM)	0.0972			0.0987	0.0997	0.255 ***			0.257 ***	0.258 ***
OIFUM × FC		0.0150					0.0484 ***			
OIFUM × LC			0.0170					0.0452 ***		
TG × LC				−0.0772 ***					−0.104 ***	
TG × FC					−0.0765 ***					−0.104 ***
Control variables	Yes	Yes	Yes	Yes	Yes	Yes	Yes	Yes	Yes	Yes
Constant	2.476	2.524	2.524	2.109	2.107	5.556 ***	5.721 ***	5.684 ***	5.069 ***	5.065 ***
N	1537	1537	1537	1537	1537	1537	1537	1537	1537	1537
R^2^	0.084	0.084	0.084	0.084	0.083	0.156	0.156	0.156	0.156	0.155

*** *p* < 0.01, ** *p* < 0.05, * *p* < 0.1.

**Table 4:** Exploratory factor analysis of risk perception of COVID-19.

Variables	Factor 1	Factor 2	Uniqueness
High infectivity	0.8901	0.0654	0.1271
Rapid fatality	0.9325	0.0789	0.1243
High mortality	0.9307	0.0816	0.1271
Personally affected	0.1761	0.7692	0.3773
Friends and family affected	0.0201	0.8	0.3596
Ordinary people affected	0.1184	0.6926	0.5063

**Table 5:** Regression model of trust in government and obtaining information channels (robustness test).

Variables	Model 3a	Model 3b	Model 3c	Model 3d	Model 3e	Model 4a	Model 4b	Model 4c	Model 4d	Model 4e
Frequency of concern (FC)	0.191 ***	0.181 ***	0.191 ***	0.191 ***	0.208 ***	0.140 ***	0.128 ***	0.140 ***	0.140 ***	0.192 ***
Level of concern (LC)	0.166 ***	0.166 ***	0.159 ***	0.183 ***	0.166 ***	0.399 ***	0.400 ***	0.389 ***	0.450 ***	0.399 ***
Trust in government (TG) (No = 0)	−0.111	−0.111	-0.112			−0.342 ***	−0.343 ***	−0.343 ***		
Obtaining information from official media (OIFOM) (No = 0)	0.119 *	0.120 *	0.119 *	0.119 *	0.119 *	−0.103 *	−0.103 *	−0.103 *	−0.103 *	−0.104 *
Obtaining information from unofficial media (OIFUM) (No = 0)	0.0824			0.0827	0.0829	0.121 **			0.122 **	0.123 **
OIFUM × FC		0.0169 *					0.0218 **			
OIFUM × LC			0.0147 *					0.0213 **		
TG × LC				−0.0199 *					−0.0605 ***	
TG × FC					−0.0204 *					−0.0605 ***
Control variables	Yes	Yes	Yes	Yes	Yes	Yes	Yes	Yes	Yes	Yes
Constant	−1.984 ***	−1.925 ***	−1.942 ***	−2.077 ***	−2.078 ***	−3.449 ***	−3.376 ***	−3.389 ***	−3.735 ***	−3.737 ***
	(0.720)	(0.721)	(0.720)	(0.717)	(0.717)	(0.679)	(0.680)	(0.680)	(0.677)	(0.677)
N	1537	1537	1537	1537	1537	1537	1537	1537	1537	1537
R^2^	0.058	0.058	0.058	0.058	0.058	0.159	0.159	0.160	0.160	0.159

*** *p* < 0.01, ** *p* < 0.05, * *p* < 0.1.

**Table 6:** Main results of models.

Variables	Cognition	Worries
Frequency of concern (FC)		+
Level of concern (LC)	+	+
Trust in government (TG) (No = 0)	−	−
Obtaining information from official media (OIFOM)	−	
Obtaining information from unofficial media (OIFUM)		+
OIFUM × FC		+
OIFUM × LC		+
TG × LC	−	−
TG × FC	−	−

## Data Availability

The data presented in this study are available on request from the corresponding author.
